# Exploring the Connection between Migraines and Pregnancy: The Impact of Physical Activity on Symptom Management

**DOI:** 10.3390/medicina60010049

**Published:** 2023-12-27

**Authors:** Milan Lackovic, Milena Jankovic, Sladjana Mihajlovic, Zagorka Milovanovic, Dejan Nikolic

**Affiliations:** 1 Department of Obstetrics and Gynecology, University Hospital “Dragisa Misovic”, Milana Tepica 1, 11000 Belgrade, Serbia; lackovic011@gmail.com (M.L.); mihajlovicobg@gmail.com (S.M.); 2 Faculty of Medicine, University of Belgrade, 11000 Belgrade, Serbia; milena.jankovic.82@gmail.com (M.J.); takibg@yahoo.com (Z.M.); 3 Neurology Clinic, University Clinical Center of Serbia, 11000 Belgrade, Serbia; 4 Clinic for Gynecology and Obstetrics “Narodni Front”, 11000 Belgrade, Serbia; 5 Department of Physical Medicine and Rehabilitation, University Children’s Hospital, 11000 Belgrade, Serbia

**Keywords:** pregnancy, migraine disorder, estrogen, physical therapy, pharmacotherapy

## Abstract

Migraine is a prevalent neurological disorder that significantly impacts the quality of life for affected individuals. The pathogenesis behind migraines is not yet fully understood, but hormonal changes, especially fluctuations in, estrogen and progesterone levels, have a significant role in the susceptibility of women to migraines. Pregnancy introduces a unique set of challenges for women who experience migraines, as they must navigate the complexities of managing their condition while safeguarding the health of both them and their unborn child. Pharmacological options for treating migraines during pregnancy are limited, and, therefore, there is a growing interest in exploring alternative approaches to migraine symptom relief and management. Physical activity during pregnancy provides a range of benefits, and it has gained attention as a potentially valuable tool for alleviating migraine symptoms in pregnant patients. This review explores the intricate relationship between migraines and pregnancy, emphasizing how physical activity and other alternative approaches may influence the frequency, severity, and overall experience of migraines during pregnancy. Through collaboration with healthcare providers and the adoption of personalized management strategies, women can strike a balance that supports both their own well-being and the healthy development of their unborn child. By examining existing research and emerging insights, we aim to provide a comprehensive understanding of the potential benefits and considerations of incorporating physical activity and other treatment options into migraine management strategies for pregnant women. Further research is needed to elucidate the specific mechanisms linking migraines, pregnancy, and physical activity, enabling the development of more targeted interventions and guidelines.

## 1. Introduction

Pregnancy is a transformative journey characterized by the joyful anticipation of a new life, which, unfortunately, may be interrupted by various physical and emotional challenges, including health-related concerns [1]. Neurological disorders during pregnancy can have varying consequences, and they are associated with significant maternal morbidity, accounting for approximately 20% of maternal deaths in the United States [2]. They may occur during all three trimesters of pregnancy, but each trimester has its own specificities and susceptibilities. Arterial–venous malformations usually occur early in pregnancy, unlike in most other neurological conditions, where they are more likely to happen later in pregnancy. The third trimester of pregnancy is associated with the highest risk of stroke, aneurysm rupture, and cerebral vein thrombosis [3]. The most prevalent neurological disorder during pregnancy is a migraine, and it affects many pregnant women worldwide [4].

Understanding the intricate relationship between migraines and pregnancy is crucial for providing expectant mothers with the best possible care. Migraine attacks during pregnancy can significantly impact a woman’s quality of life and may even raise concerns about the potential risks to both maternal and fetal health [5]. Additionally, the management of migraine symptoms becomes even more complex during pregnancy due to restrictions on medication use and the need to balance relief with the safety of the developing fetus [6]. 

While pharmacological options for treating migraines are limited during pregnancy, there is growing interest in exploring alternative approaches to symptom relief and management. Physical activity, including exercise and relaxation techniques, has gained attention as a potentially valuable tool for alleviating migraine symptoms in pregnant patients. However, the relationship between physical activity and migraines during pregnancy is complex and not yet fully understood [7,8].

This review aims to shed light on the interplay between migraines and pregnancy, emphasizing how physical activity and other alternative approaches may influence the frequency, severity, and overall experience of migraines during this transformative phase of life. By examining existing research and emerging insights, we aim to provide a comprehensive understanding of the potential benefits and considerations of incorporating physical activity and other treatment options into migraine management strategies for pregnant women. Such knowledge can empower healthcare providers and pregnant individuals alike to make informed decisions that promote both maternal well-being and the healthy development of the fetus.

## 2. Migraine Impact on Women’s Health and Life Quality 

Migraines are complex, genetically determined conditions that are characterized by recurrent, moderate to severe, and usually unilateral headaches, which are commonly accompanied by vomiting, nausea, and sound and light sensitivity [9].

According to the third edition of the International Classification of Headache Disorders (ICHD-3), migraines are divided into six categories: migraine without aura (MWA), migraine with aura (MA), chronic migraine, complications of migraine, probable migraines, and episodic syndromes that may be associated with migraine [10]. Most of these categories can be subcategorized, but from the clinical aspect of view, MWAs appear to be the most relevant. MWAs are primarily associated with hormone level changes and are accountable for approximately 80% of all cases of migraines [11].

More than 15% of the world’s population report some of migraine attack symptoms at some point of their life [12]. Notable sex dimorphism in migraine prevalence is present worldwide (Table 1). Even though migraines can affect individuals of any gender and age, women are disproportionately affected in significantly higher proportions and prevalence across all age groups [13]. The peak prevalence of migraine onset among women is the reproductive age, with hormonal fluctuations playing a significant role in migraine onset and symptom severity [14]. Furthermore, functional as well as structural differences in the central nervous system have been found between women and men with migraines by magnetic resonance imaging [15]. 

Before puberty, migraines are two to three times more prevalent among girls, and after puberty, migraines are three to four times more frequent in adult women compared with men [16]. According to the Global Burden of Disease study, they are categorized as the fourth leading cause of years living with disability (YLD) for women, unlike for men, among whom they are categorized as the eighth leading cause of YLD [17]. Migraine was estimated to have caused more than 45 million cumulative years of YLDs, exceeding USD 19 billion a year in the US in indirect costs alone [17,18]. 

The teens and early twenties are the usual age for the onset of migraines, and peak prevalence is usually reached in the early forties, with a tendency for migraine symptom reduction after menopause. According to the age prevalence distribution, menstruation appears to be one of the key factors initiating the vicious cycle of migraine symptoms. More than 50% of women affected by migraines report an association between menstruation and migraine onset, describing it as most likely to occur in the time range from two days before the menstruation until the first three days of the menstrual cycle [19]. In favor of the hormonal impact on migraine onset, epidemiological data reveals that in prepubertal children aged 7–9 years, there is no change in migraine prevalence. First, but still insignificant, differences appear among children aged 10–12 years (5.4% of girls compared with 3.9% of boys) [20], the age that usually coincides with the occurrence of menarche. 

Starting from puberty and the onset of intensive sex hormonal changes, the overall prevalence of migraines begins to increase, affecting primarily girls. Up to 20% of women suffering from migraines report their first migraine attack at the age of menarche [21]. 

Migraines have a bimodal prevalence pattern; they reach their initial peak at the age of 35, and the second one at the age of 50. Afterwards, the frequency and symptoms manifested start to decrease [22]. Conversely to migraine prevalence, migraine peak incidence in women is observed between the age of 20 and 24, and in men between the age of 15 and 19 [23].

Despite significant differences in the migraine occurrence between women and men, there seems to be no sex-related difference in migraine attack frequency between the genders. Both women and men appear to suffer from migraine-related symptoms on average one to four days monthly [24]. Migraine studies have proved that migraine-associated symptoms significantly impact everyday life activities for both men and women. They are responsible for family and relationship disruptions and educational, career, and financial problems among women and men in similar ratios [25]. 

Both men and women experience similar clinical manifestations. In the case of migraine with aura, an attack typically consists of four stages: prodrome, aura, attack, and post-drome. The prodrome phase usually begins one or two days before an upcoming migraine. Most commonly, patients experience it as constipation, changes of mood, neck stiffness, food cravings, fluid retention or increased urination. Aura might occur before or during a migraine attack. An aura is usually visual, but other sensations might be involved as well. Typically, they evolve gradually, and can last for up to 60 min. Migraine attacks usually last between 4 and 72 h, while the frequency of their occurrence widely ranges. A migraine attack is characterized by pain accompanied by nausea and vomiting. Throbs are typical pain manifestations, along with sound and light sensitivity, but smell and taste sensitivity might occur as well. A migraine postdrome phase is not uncommon. In the postdrone period, patients might experience vide range of symptoms, such as exhaustion, difficulty concentrating, mood changes, digestive issues, neck stiffness, and aches [26]. 

Patterns are one of the main characteristics of migraines and they consist of attacks, remissions, and relapses. Compared with women, men appear to have a more favorable pattern set, since the remission period for men seems to last longer [27]. 

Migraine can be classified as active and inactive as well. The occurrence of grain migraine symptoms within the last year is diagnostic for an active migraine. Study results coming from Germany imply that women of older age are less commonly affected by active migraines compared with men. The prevalence of inactive migraine in Germany is 25.7% for women, compared with only 16.5% for men aged 60 or more. This discrepancy changes the balance of migraine prevalence between men and women once again and suggests that female-sex-hormone depletion interferes significantly with the migraine prevalence [28]. 

Along with migraines, tension-type headaches (TTHs) are the most common primary headaches. During pregnancy, hormonal changes may influence the clinical manifestation, and due to symptom overlaps, it is not always easy to distinguish migraine from an episodic TTH. Unlike migraines, TTHs usually tend to be bilateral in location, and they typically cause a steady ache, but, most importantly, they are not correlated with any adverse pregnancy outcome [29]. TTHs do not worsen with routine physical activity. Lifestyle interventions are effective in headache prevention and treatment, but pharmacotherapy remains the mainstay of clinical management [30,31]. 

## 3. Migraines and Hormones: How Does the Pregnancy Interfere?

The pathogenesis underlying migraines, a complex neuromuscular disorder, is not yet fully understood. Undoubtedly it is mediated by hormones, and hormonal differences, especially fluctuations in estrogen and progesterone levels, have a significant role in the susceptibility of women to migraines [32]. Determining the exact pathophysiology mechanism that explains migraines is a matter for extensive research. Most of the results are based on animal models [23], and due to the lack of studies performed in humans, especially males, it remains a challenge to draw conclusions [24]. Aside from estrogen and progesterone fluctuations, it is known that inflammatory mediators affecting the trigeminovascular system have a prominent role in migraine pathogenesis as well as in sex-related differences in migraine occurrence [33]. Prolactin, a pituitary-derived hormone, and oxytocin, a hypothalamic neuropeptide, have become the recent focus of research interest, since it appears that they interfere with the migraine onset and symptom development [34,35]. Prolactin and oxytocin are nociceptive triggers that are involved in opposite ways. Prolactin is a pronociceptive agent, while oxytocin appears to have an antinociceptive role [33]. Changes in prolactin and oxytocin levels are closely related to pregnancy and symptom management; therefore, their potential use as agonists and antagonists creates a promising possibility for targeted migraine therapy [33].

**Figure 1:**
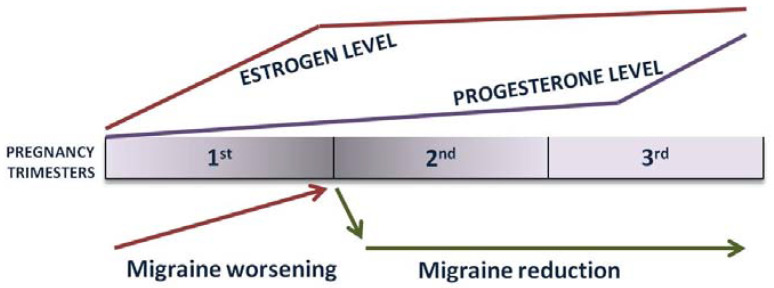
Estrogen fluctuations and migraine course during pregnancy.

Pregnancy, marked by dramatic hormonal changes, poses a unique challenge for women with pre-existing migraines, necessitating a careful balance between managing their condition and ensuring the well-being of the developing fetus. Estrogen, progesterone, and other hormonal changes; disrupted sleep; stress; nausea; and dehydration during pregnancy can influence the frequency and severity of migraines. While some women experience migraine symptom relief during pregnancy, others may experience an exacerbation of their symptoms, particularly in the first trimester of pregnancy. MWAs have a general tendency to improve compared with MAs, which are also more likely to occur during de novo pregnancy [36]. The first trimester of pregnancy is the time when estrogen levels rise; therefore, during this stage of pregnancy, it is possible for some women to experience transient symptom worsening. The second and the third trimesters are usually known for their tendency for symptom relief, and a nearly 90% symptom reduction is expected during this time of pregnancy [37]. During this stage of pregnancy, hormone levels are stable, estrogen and endogenous opioid levels are elevated, and, most importantly, there are no more hormone fluctuations, which eliminates one of the major attack triggers (Figure 1) [36].

Acute migraine affects pregnancy outcomes in several different ways. A retrospective study conducted in the US revealed that in a case of severe acute migraine during pregnancy, more than half of the study participants were more likely to develop at least one adverse pregnancy outcome, such as pre-eclampsia, preterm delivery, or low birthweight [38]. Therefore, patients experiencing acute migraine symptoms must be considered high-risk patients, and in a case of a new headache onset, other pregnancy-related complications, such as a hypertensive disorder of pregnancy, should undoubtedly be excluded first [39]. 

**Figure 2:**
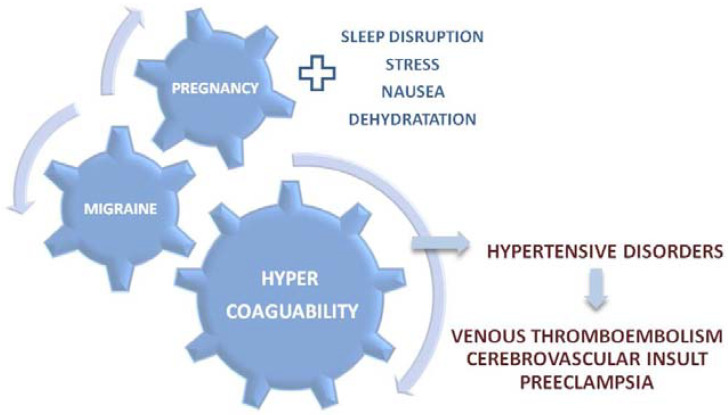
Hypercoagulability as a crosslink between migraine, pregnancy, and maternal/fetal morbidity.

Pregnancy and migraine have another common characteristic, hypercoagulability (Figure 2). During pregnancy, hypercoagulability is a significant risk factor for some of the most serious cardiovascular incidents, including venous thromboembolism and cerebrovascular insult [36]. Furthermore, interference between hypercoagulability, cardiovascular disorders, and pre-eclampsia present another significant risk factor for the maternal, fetal, and neonatal morbidity and mortality [40]. Among patients with persistent migraine symptoms during pregnancy, symptom deterioration is associated with a 13-fold higher risk of a hypertensive disorder in pregnancy compared with the patients who experience migraine symptom improvement, or whose symptoms are remitted [41].

## 4. Physical Activity—Are There Two Sides to the Coin?

### 4.1. Physical Activity and Pregnancy

**Figure 3:**
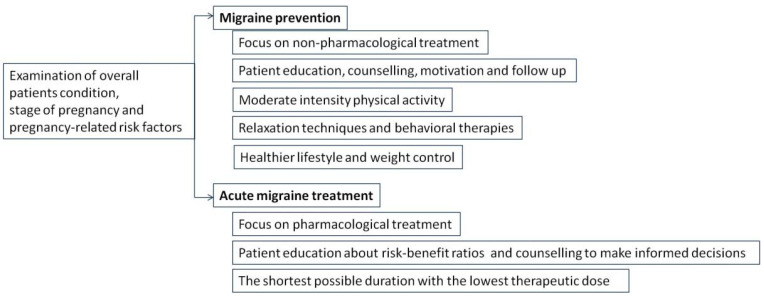
Recommendations for migraine management during pregnancy.

It appears that pharmacotherapy has only a moderate impact in migraine management, and pharmacological treatment options available for pregnant migrainous women seem to be very limited [42]. Non-pharmacological treatment, such as physical activity (PA), is usually recommended for mild migraine sufferers during pregnancy, and it is usually the therapy of choice for patients suffering from migraines during pregnancy. In order maximize the benefits of such a treatment, healthcare providers should motivate women of reproductive age to adopt healthy lifestyles that include regular PA, and migrainous women should be encouraged to engage in regular PA before becoming pregnant [43]. Furthermore, since pregnancy is recognized as a “teachable moment”, pregnant women are usually motivated to adapt healthy behavior changes, and physicians should take this opportunity as an advantage to motivate, encourage, and support their patients (Figure 3) [44]. Moderate-intensity PA is strongly recommended during pregnancy, and, according to the Royal College of Obstetrics and Gynecologists (RCOG), it should last at least 150 min per week. It may include climbing stairs, yoga, swimming, carrying grocery shopping bags, cycling, dancing, using the treadmill, or simply taking a walk [45]. 

PA during pregnancy provides a range of benefits [46,47]. Regular exercise is helpful in stress reduction, better sleep, improved circulation, and weight management, all of which can be important aspects of migraine management [48]. When introduced during pregnancy for the first time, PA level must be started gradually. The patients’ overall condition, stage of pregnancy, as well as pregnancy-related risk factors should be cautiously analyzed before any type of exercise is proposed to a patient. The RCOG strongly advises patients not to engage in any activity which may involve the risk of falling or suffering a potential abdominal injury; it also stresses the danger of high-altitude environments and hot temperatures [45]. 

### 4.2. Physical Activity and Pathophysiology

PA interferes with inflammatory mediators, and it suppresses the levels of some stress hormones, including cortisol and growth hormone [49]. The migraine neuroinflammatory model implies a significant role of elevated inflammatory markers, cytokines, and adipocytokines in a migraine setting. Regular aerobic exercise has a suppressive effect on inflammatory mediators, and, eventually, it may lead to a suppression of the intensity, frequency, and duration of migraine attacks [50]. PA may activate several pathways, including endogenous opioid and cannabinoid systems, brain-derived neurotrophic factor, as well as some behavioral and psychological factors, which may eventually attenuate migraine symptoms. Hence, PA is a potentially effective strategy platform in migraine treatment and prevention [8]. Furthermore, PA stands as a promising, effective, and feasible complimentary or augmentative therapy for suboptimally treated migraineurs [50]. 

Integrated therapy modalities provide the highest level of care in medicine and therefore, behavioral therapy, combined with PA and pharmacotherapy, should be considered indispensable whenever possible. Cognitive behavioral therapy, relaxation, and biofeedback therapy interfere with lifestyle habits involved in the transformation of episodic to chronic migraines, a disabling condition that affects a substantial proportion of patients [51]. Relaxation exercises, such as prenatal Pilates or gentle stretching, may reduce everyday tension and pain and promote the overall wellbeing and quality of life during pregnancy, and these could be incorporated in pregnant women’s daily routine [52].

The majority of studies underline the significance of PA in migraine symptom management and acknowledge the role of PA as a relevant resource for migraine treatment. However, some authors did report ambiguous conclusions regarding the effectiveness of PA [53]. Indeed, PA was identified as a migraine-triggering factor by some authors, but this was primarily associated with intensive PA [54]. In a study published by Varkey et al., a cycle ergometer test was used until exhaustion to provoke migraine attacks. This group of authors concluded that maximal aerobic exercise may be a migraine-attack trigger, but they emphasized that it does not necessarily lead to a migraine attack [55]. Controversially, low PA, defined as less than 30 min of PA per week, was identified by Lebedeva et al. as a statistically significant risk factor for migraine development in women, but not in man [56]. 

A possible explanation behind PA as a migraine triggering factor is related to an increase in nitric oxide (NO) synthesis and to the consequential changes in blood flow [57,58]. PA is associated with intrinsic NO production; it augments endothelium-dependent vasodilatation [57,58], leads to cerebral arterial dilatation, and eventually it may act as a triggering factor for vascular headaches and migraines [59]. Additionally, it is a well-known fact that nitroglycerin and other drugs that generate NO are migraine triggers in the majority of migraine patients [60]. 

Even though some types of PA may be a triggering factor for migraines [48], regular PA has a prophylactic effect [48]. Altered thresholds triggering migraine attacks are the most likely cause of reduced attacks among migrainous people who exercise regularly [61]. Accumulated scientific evidence now provide more insight into the true connections between migraines and PA. Despite the fact that a systemic review did find moderate-quality evidence associating PA with a decreased number of migraine days, it could not draw clear conclusions regarding migraine attack duration or pain intensity [62]. Luckily, a more recent meta-analysis showed a clinically significant difference in pain intensity between patients engaged in regular PA and those who are not [63].

Finally, individuals with headaches tend to be less physically active; therefore, it remains challenging to establish clear connections between migraines and PA, since migraines are a well-known disabling condition that is commonly associated with limited social interferences and PA [64]. Cognitive decline, anxiety, depression, and sleep deprivation are frequently attributed to people suffering from migraines [65], and the advantages of PA may be used for symptom control and the management of these comorbidities as well [50].

## 5. Other Lifestyle Choices and Interventions 

**Figure 4:**
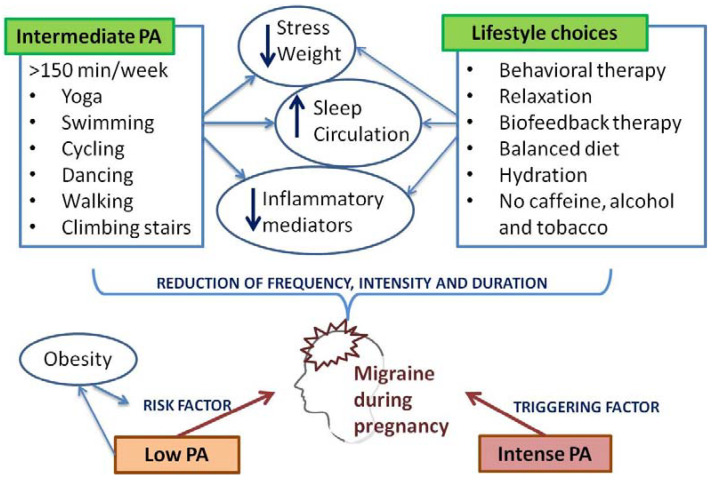
Effects of physical activity and lifestyle choices on migraine during pregnancy.

PA requires patients’ committed involvement, and it is often time consuming. Other lifestyle interventions and changes in daily habits, including a balanced daily calorie intake, hydration with non-caffeinated fluids, stress reduction, and complete alcohol and tobacco avoidance, may be an effective alternative for migraine frequency reduction as well (Figure 4) [66]. Most of these recommendations and interventions are the actual requirements of pregnancy, and their incorporation into pregnant migraineurs’ daily life could at least partially explain the tendency of frequency and severity of migraines to decrease during pregnancy [67].

Obesity is a well-known risk factor for pregnancy outcomes and for the well-being of the offspring [68,69], but it is also a risk factor associated with increased migraine prevalence and frequency [70,71]. There is no specific diet prescribed for migraine sufferers, but for all obese patients, a weight-loss diet is recommendable [72]. Diet during pregnancy should always be strictly balanced, and the required intake of micro- and macronutrients, as well as a minimum of 1600 kcal/day, ought to be provided to all patients [73]. The Mediterranean diet provides the nutritional requirements of pregnancy. In addition, it may result in weight loss, has several cardiovascular benefits, and it is the optimal diet to consume in pregnancy [74]. Other dietary regimes, such as the elimination diet, includes the identification and elimination of the migraine-provocative ingredient, and it may be an effective alternative as well [75]. 

The US Institute of Medicine (IoM) recommendations are based on age and gender. According to the IoM, water intake should range from 2.7 to 3.7 L, or nine to thirteen cups, per day [76]. IoM recommendations are in agreement with the requirements in pregnancy for water intake, which range from eight to twelve cups per day [77]. Adequate hydration accounts for balanced ion concentrations and plasma osmolality, which are important for migraine control and management [78]. Caffeine use may lead to an increase in migraine frequency, but its withdrawal may induce headaches as well. Migraine sufferers who choose to continue drinking caffeinated beverages should keep their daily intake consistent and should not exceed a daily consumption of 200 mg of caffeine daily [79]. Pregnant women are advised not to take more than 200 mg of caffeine per day as well. According to the American College of Obstetricians and Gynecologists (ACOG), this moderate caffeine consumption is not a major contributor for a preterm birth or a miscarriage [80].

There is a well-known notion that alcohol consumption may trigger different types of headaches, but without taking into account individual dispositions and different cultural backgrounds, it is not justified to advise all patients with headaches to abstain from alcohol completely [81]. In several different pathways, alcoholic beverages may cause a migraine attack that includes an inflammatory pathophysiology mechanism, dehydration, vasodilation, toxicity, and the release of histamine, sulfites, tyramine, flavonoids and 5-HT [82]. Excessive and chronic use of alcohol is undoubtedly associated with poor migraine control and pregnancy outcome, but some reviews did find a beneficial effect of low doses of alcohol on migrainous patients [82]. Smoking can trigger migraine attacks, and this risk is dose-dependent on the total number of cigarettes smoked per day [83]. Aside from migraine management, behavioral interventions targeting alcohol and smoking cessation during pregnancy and the preconceptional period are crucial in avoiding poor pregnancy outcomes and the inevitable consequences for the offspring [84].

Neuronal and hormonal changes provoked by stress may have multiple relationships with migraines, but the exact mechanism by which stress causes migraine attacks remains unclear [85]. Sleep deprivation, insomnia, and a lower quality of life are well-known stress provokers, and by eliminating these primary causes of stress, the migraine incidence and symptom management may effectively be controlled [86].

Another helpful resource in migraine management may be the use of smartphones with installed apps and electronic diaries. A diary of attacks can give provide insight into the migraine frequency, duration, and potential triggers, and lead to better migraine management [87]. 

In Table 2, the types of physical activities and the outcomes associated with pregnancy are presented.

## 6. Pharmacotherapy 

**Figure 5:**
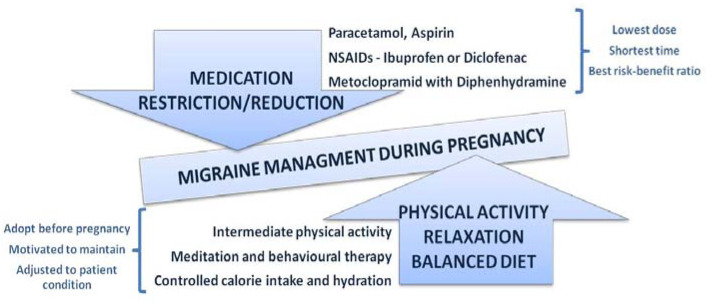
Balancing migraine management in pregnancy.

Prevention, early diagnosis, regular follow ups, and non-pharmacological treatment options are the cornerstones of migraine treatment whenever possible (Figure 5) [98]. Acute migraine often requires pharmacological treatment in order to reduce the attack frequency and duration and to provide the optimal treatment. Whenever a new drug is prescribed to a pregnant woman, treatment should be tailored to the shortest possible duration with the lowest therapeutic dose, and a risk–benefit ratio should be presented and clarified [99]. 

Acetaminophen (paracetamol), metoclopramide, diphenhydramine, non-steroidal anti-inflammatory drugs (NSAIDs), and NSAID-triptan combinations are the usual pharmacotherapeutic options available for acute migraine attacks [100,101]. For headaches and pain management, paracetamol and NSAIDs are usually the most appropriate, although weaker opioids administrated over the short term are generally considered to be safe in pregnancy as well [102]. 

Paracetamol is recommended as the first choice of treatment for acute migraine during pregnancy, but the potential side effects of paracetamol use during pregnancy must not be neglected [103]. According to some study results, paracetamol might negatively interfere with the maternal hormones involved in embryonic brain development, or it might induce oxidative stress reaction, leading to neurotoxicity and to irreversible neuron loss [104].

NSAIDs are the second line of treatment, with ibuprofen and diclofenac being the most commonly used NSAIDs. The use of NSAIDs should be avoided for an extended period of time during the first and the third trimesters of pregnancy, since it is associated with certain risks and contraindications [105]. 

Although metoclopramide and diphenhydramine are not typically used for prolonged amounts of time, their use is considered safe during pregnancy. In pregnant women, metoclopramide administered with diphenhydramine effectively relieves migraine headaches when paracetamol fails [106].

Triptans and ergot derivates have been associated with an increased risk of adverse pregnancy outcomes, including prematurity, congenital abnormalities, miscarriages, and low birth weight [107], and only patients with severe attacks who are poor responders to other treatment options should consider the triptan approach [108].

Opioids are extremely rarely prescribed during pregnancy, and only for severe and intractable migraine pain. Opioid pain relievers can be addictive for both the mother and the newborn; their use in early pregnancy increases the risk of congenital abnormalities, and they are associated with preterm birth and fetal growth restriction [109]. 

Specially designed to prevent migraines, Gepants were the first oral agents ever introduced in migraine treatment. Gepants are CGRP (calcitonin-gene-related peptide) antagonists with proven activity in migraine prevention and treatment. Along with neurokinin A, NO, and substance P, CGRP leads to vasodilatation, protein extravasation, and sterile inflammation, causing nociceptor activation. Despite its effectiveness, the use of Gepants remains controversial due to possible long-term side effects, primarily those associated with vascular hemodynamic impairment [110]. 

Low-dose aspirin use during pregnancy has proven its benefits for some pregnancy-related disorders, and despite insufficient evidence proving its usefulness in migraine treatment during pregnancy, a 100 mg dose of aspirin seems, at least, to be harmless [111].

## 7. Conclusions

Migraines, pregnancy, and physical activity intersect in a complex web of physiological and lifestyle factors. Pregnant women with migraines face the challenge of managing their condition while reaping the benefits of physical activity during this critical period. Through collaboration with healthcare providers and the adoption of individualized management strategies, women can strike a balance that supports both their own well-being and the healthy development of their unborn child.

Physical activity may be a reliable treatment option for pregnant women, but it is essential to approach physical activity gradually to avoid triggering migraines. In terms of future perspectives, ongoing research continues to explore the relationship between migraines, physical activity, and potential therapeutic interventions. Advances in personalized medicine may lead to tailored approaches for managing migraines based on individual’s specific triggers and responses. Engaging in regular physical activity has been linked to a potential reduction in the frequency and severity of migraines; however, this relationship is complex and varies among individuals. Some individuals find exercise helpful, while others may experience migraines that are triggered by physical activity. Therefore, further research is needed to understand the mechanisms behind this connection and to develop personalized recommendations for migraine management through physical activity. 

## Figures and Tables

**Table 1:** The sex dimorphism in migraine patients.

Migraine Characteristics		Female	Male
Peak incidence (age)		20–24	15–19
Peak prevalence		Reproductive age	Life-long
Prevalence ratio	Before puberty	2–3	1
	After puberty	3–4	1
Inactive migraine prevalence		25.7% after the age of 60	16.5% after the age of 60
Years living with disability (YLD)		4th leading cause	8th leading cause
Remissions		Shorter	Longer
Clinical manifestations		Similar	
Attack frequency		1–4 days per month	
Impact on quality of life		Similar	

**Table 2:** Type of physical activity and outcomes associated with pregnancy.

References	Type of Physical Activity	Outcomes
Ref. [88]	Lumbar stabilization and stretching	Pain Disability Postural control Muscle activity
Ref. [89]	Aerobic and strength training Pregnancy-specific floor exercises	Postpartum depression
Ref. [90]	Aerobic exercise Muscle strengthening Coordination and balance exercise Stretching exercises Pelvic floor strengthening Relaxation	Weight gain Gestational diabetes
Ref. [91]	Water exercise	Health-related quality of life
Ref. [92]	Pilates exercises	Childbirth outcomes
Ref. [93]	Aerobic exercise	1-month infant neuromotor skills
Ref. [94]	Physical conditioning program (warming up, cardiovascular exercise, strengthening exercise, coordination and balance exercise, pelvic floor exercise and stretching and relaxation)	Maternal weight gain Fetal cardiac function
Ref. [95]	Supervised exercise training	Sleep Sedentary time
Ref. [96]	Aerobic exercise	1-month infant morphometry
Ref. [97]	Aerobic exercise	Fetal cardiac function and outflow
